# Concentration of potentially toxic elements in fillet shrimps of Mediterranean Sea: Systematic review, *meta*-analysis and health risk assessment

**DOI:** 10.1016/j.fochx.2024.101206

**Published:** 2024-02-07

**Authors:** Trias Mahmudiono, Zahra Esfandiari, Ali Zare, Mohammadmahdi Sarkhoshkalat, Fereshteh Mehri, Yadolah Fakhri

**Affiliations:** a Department of Nutrition, Faculty of Public Health, Universitas Airlangga, Surabaya, Indonesia; b Nutrition and Food Security Research Center, Department of Food Science and Technology, School of Nutrition and Food Science, Isfahan University of Medical Sciences, Isfahan, Iran; c Food Health Research Center, Hormozgan University of Medical Sciences, Bandar Abbas, Iran; d Department of Mechanical Engineering, Islamic Azad University, Mashhad, Iran; e Nutrition Health Research Center, Center of Excellence for Occupational Health, Research Center for Health Sciences, School of Public Health, Hamadan University of Medical Sciences, Hamadan, Iran

**Keywords:** Potentially toxic elements, Marine foods, Food Safety, Shrimps, Mediterranean Sea, Risk Assessment

## Abstract

In this study, an attempt was made to *meta*-analyzed the concentration of potentially toxic elements (PTEs) in shrimps tissue of Mediterranean Sea and health risk of consumers was estimated. Search was conducted in international databases includes Scopus, PubMed, Embase, Science Direct and Web of Science from 1 January 2010 to 20 July 2023. The random effects model used to *meta*-analysis of concentration of PTEs in shrimp in subgroups. In addition, non-carcinogenic and carcinogenic risks for adults and children were calculated using target hazard quotient (THQ) and cancer risk (CR). Meta-analysis concentration of PTEs in shrimps was conducted using random effects model based on country subgroups. The rank order of PTEs based on mean (pooled) level in fillet of shrimps was Fe (15.395 mg/kg-ww) > Zn (10.428 mg/kg-ww) > Cu (6.941 mg/kg-ww) Pb (5.7 mg/kg-ww) > Ni (1.115 mg/kg-ww) > As (0.681 mg/kg-ww) > Cd (0.412 mg/kg-ww) > Hg (0.300 mg/kg-ww). THQ level in adults and children due to Cd and Pb in Italy was higher than 1 value. THQ level in adults and children due to Cu, Ni, Fe, Zn and inorganic As was lower than 1 value. CR due to inorganic As in Greece and Türkiye for adults and children was higher than 1E-6 value. Therefore, it was recommended to continuously monitor and reduce the concentration of PTEs in shrimps in Italy, Greece and Türkiye, especially.

## Highlights

• The rank order of PTEs was Fe > Zn > Pb > Ni > As > Cd > Hg.
• THQ in adults and children due to Cd and Pb in Italy was higher than 1 value.
• CR due to inorganic As in Greece and Turkey for adults and children was higher than 1E-6 value.

## Introduction

Regarding with the expansion of anthropogenic activities, e.g., industrial, agricultural and mining happenings, the level of some contaminants such as potentially toxic elements (PTEs) has increased in different sources including environment, water, soil, and food in recent years (Akhtar et al., 2021, Assad et al., 2023). Environmental pollution (Li et al., 2020, Ma et al., 2022, Tang et al., 2023, Zhang et al., 2020, Zhao et al., 2023, Zhao et al., 2023) in soil (She, Gao, & Shi, 2023), air, water resources, food such as meat (Özlü et al., 2023), rice (Takooree et al., 2023), wheat (Bai et al., 2023, Han et al., 2023) has increased over decades. These pollutants includes microbial (Ahmad et al., 2023, Kamal et al., 2023, Zhao et al., 2023, Zhao et al., 2023), mycotoxins (Basso et al., 2023, Tenge et al., 2022) and heavy metals (Zoghi et al., 2022) that cause various diseases (Guo et al., 2023). Furthermore, these elements certainly exist in environment because of natural disaster, weathering, and volcanic reactions (Braune et al., 2015). PTEs have larger density and higher atomic mass. These types of contaminants such as mercury (Hg), and nickel (Ni), arsenic (As), lead (pb), cadmium (Cd) are non-degradable with the ability of accumulation in different tissues of organisms (Rahman and Singh, 2019). PTEs inhibit cellular organelles and mechanisms includes lysosome and cell membrane. Interaction of PTEs with DNA cause protein damaging and conformational alterations with the consequences of cycle modulation, carcinogenesis or apoptosis (Al Osman et al., 2019). Eexposure to high level of metallic Hg may result in skin rashes, nausea, lung damage and increasing the blood pressure. The toxicity signs of organic Hg are depression, tremors, headache, and fatigue (Atti et al., 2020). Stomach discomfort, hypertension, Headaches, insomnia, fatigue and dizziness are symptoms of Pb exposure (Guzzi et al., 2021). Some negative impacts are mentioned through contact with Ni including kidney and heart issues, allergies, nasal and lung cancer. The molecular mechanisms of Ni for toxicity are not yet known (Valko et al., 2005). However, it is supposed that oxidative stress and mitochondrial malfunction have important role in its toxicity (Ugulu, 2015). As has more complex chemical structure and can be present both as inorganic and as a range of organic As forms. The large differences in toxicity are reported for As compounds. Inorganic As is categorized as class I carcinogenic type, and its determination is important for assessment of food safety (Joint et al., 2011). Organic form of As “arsenobetaine” exists in fish and crustaceans and is considered as non-toxic. Around 90 % of total As in seafoods is arsenobetaine (Luvonga et al., 2020). Cd has no biological role in organisms, and causes adverse health effects even at low level (Olmedo et al., 2013).

Contamination of water with PTEs is considered as one of the most serious environmental worries related to plants, animals, and human health (Wang et al., 2021). According to various reports, chronic poisoning with PTEs in aquatic animals has adverse health effects such as growth reduction and reproduction and histopathological effects. PTEs are first absorbed by phytoplankton, bacteria, fungi and small organisms, then eaten by larger organisms such as shrimps and finally enter the human body (Soultani et al., 2021). Currently, due to the ever-increasing growth of the world's population and the increase for protein requirements around the world, especially with the discovery of the possible negative effects caused by the consumption of red animal proteins, the demand for the consumption of shrimps and marine animals is rising (Boyd et al., 2020). So that, aquaculture has been the fastest-growing food production section with a growth rate of 8 %, and supplying 49 % of global seafood demand annually (FAO, 2014).

Shrimps are introduced as important nutritional, economic and cultural components of the world’s food supply (Boyd et al., 2020). Shrimps are included just 4 % of the world fisheries by weight. However, their financial value is 11 % of total commercial fisheries. Shrimps aquaculture has greatly increased in recent years, so that it is more than double that of shrimps fisheries in production and economic value (Bauer, 2023). Although some of the contaminants entered into water resources are significantly back to the original state and out of the external cycle and endanger the ecosystem, but they do not pose a serious threat to the lives of marine animals. However, PTEs are resistance to biological changes after entering the environment with the ability of continuation of their cyclic movement in the life cycle (Luo, 2022, Al Osman et al., 2019).

Shrimps exposure to PTEs occurs by direct contact with polluted environment or cooking processes and accordingly enters to human body through food chains (Siripongvutikorn, 2023, Kalahal, Gavahian, & Lin, 2024). The Mediterranean Sea covers an area of about 2,500,000 km2, representing 0.7 % of the global ocean surface. Different countries have a coastline on the Mediterranean Sea such as Spain, Monaco, France, Bosnia and Herzegovina, Italy, Slovenia, Greece, Croatia, Lebanon, Montenegro, Albania, Syria, Türkiye, Palestine, Egypt. Many activities such as offloading goods, ballasting and fueling occurs around this sea. So, it is necessary to assess and monitor the exposure of human through consumption of shrimps contaminated with PTEs in this area (Abd-Elghany et al., 2020).

For example, in an Egyptian study, the average levels of As and Pb either in raw or cooked (boiled or grilled) crab and shrimps samples analyzed were higher than WHO/FAO guidelines, which set 0.15 and 0.20 mg/kg dry weight for As and Pb, respectively. In other hands, the average of Cd were below than WHO/FAO standard (0.1 mg/kg dry weight) in boiled crabs, grilled crabs and grilled shrimps, although it was higher than such limit in raw and boiled shrimps (Abd-Elghany et al., 2020). In another study performed in Türkiye, it was found that the level of PTEs including Cd, Pb, Cu, Zn, and Fe in muscle tissue of shrimps was less than the authorized range of WHO and FDA limit (Olgunoğlu et al., 2015). Several studies have been conducted in recent years on the concentration of PTEs in shrimps of Mediterranean sea (Dökmeci et al., 2014, Olgunoğlu et al., 2015, Oudainia et al., 2023, Özden, 2010, Skordas et al., 2022, Soultani et al., 2021), but there was no *meta*-analysis study on the concentration of PTEs in shrimps of Mediterranean. The main aims of current study were *meta*-analysis concentration of PTEs in shrimps of Mediterranean and health risk assessment.

## Materials and method

### Searching strategy

The systematic review was conducted according to PRISMA protocol (Fu et al., 2023, Khanverdiluo et al., 2021, Moher et al., 2015, Zeng et al., 2017). The search was performed by two authors (A.ZA and Y. FA) in databases includes Scopus, PubMed, Embase, Science Direct and Web of Science from 1 January 2010 to 20 July 2023. The English keywords were obtained based on published preliminary papers and medical subject headings (MeSH Terms). Keywords were including “**Potential hazard element**” OR “**Potential toxic element**” OR **elements**“ OR ”**Potentially toxic elements** “ OR” **Trace metals**“ OR ”**Toxic elements**” AND ***Caridea*“ OR shrimps**” **OR Marine Foods**“ OR **”*Caridean Shrimps***“ **OR ”Marine food“ AND ”Mediterranean Sea“ OR ”Great Sea“.** The search was performed by two authors (A.ZA and F.ME) separately according to the inclusion and exclusion criteria. All papers were collected using EndNote software version 8. Duplicate papers were removed and papers were screened based on the title and abstract. After downloading the papers, the full text was read and the when met our inclusion criteria were entered into the data extraction stage. If there was disagreement on the selection of papers between the authors (A.ZA and F.ME), The final comments of the corresponding author were the criterion for selecting or removing of paper (Gao, 2023, Zamanpour, Noori, Yancheshmeh, Afshari, & Hashemi, 2023). References list of retrieved papers was screened to explored missed relevant papers.

### Inclusion/exclusion criteria and data extraction

Our inclusion criteria were including full texts presented as English text; analysis of PTEs in fillet shrimps in Mediterranean Sea, use reliable detection method, present level statistics (average, range, standard deviation). Book chapters, Books, review papers, thesis, letters to editors and conferences were excluded (Huang et al., 2022, Tian, 2023, Aranega & Oliveira, 2022). The species of shrimps, country, Statistics level (methyl-Hg, inorganic As, Cd, Ni and Pb, Cu and Zn), includes mean, standard deviation, and analysis technique were extracted. The total As was converted to inorganic As using a 5 % coefficient in the some investigations that presented total As in shrimps (Fakhri and Mousavi, 2020, Vu et al., 2017, Yadolah and Mansour, 2021, Wang, 2023).

### Meta-analysis

Meta-analysis of level of PTEs in shrimps was conducted by mean and standard error statistics. The *I^2^* statistic was applied to detect the heterogeneity; when I^2^ is higher than 50 %, random effects model was used to estimate the pooled (mean) effect size. The *meta*-analysis of data was conducted by Stata Version 14.0 (College Station, TX, USA).

### Health risk assessment

The chronic daily intake due to ingestion of shrimps content of PTEs was calculated by below equation (Einolghozati et al., 2022, Qin et al., 2023):

(1) $$CDI=\frac{C\;\times \;IR\;\times \;ED\;\times \;EF}{BW\;\times \;AT}$$

In this equation, CDI is chronic daily intake; C, level of PTEs in shrimps; IR is ingestion rate; ED is exposure duration (children and adults are 6 and 70 y, respectively); EF is exposure frequency (350 d/y); and BW, body weight (children and adults are 20 and 70 kg, respectively). AT is the average lifetime (noncarcinogenic risk for children (2190 d) and adults (25550 d) and carcinogenic risk: children and adults is 25,550 d).

On mean, 17.5 % of the consumption of marine foods is dedicated to shrimps (Fan et al., 2022, Yu et al., 2020). Mean ingestion rate of shrimps in countries was presented in **Appendix 1**. The tolerable daily intake for Pb is equal to 0.0036 mg/kg-d (FAO/WHO, 2011). The oral reference dose (RfD) for inorganic As, Ni, methyl Hg, Cd, Cu and Zn are 0.0003, 0.11, 0.0001, 0.001, 0.04 and 0.3 mg/kg-d (EPA, 2021). The non-carcinogenic risk of PTEs was calculated by below equation (Gavahian, 2023, Ghane et al., 2022, Xiong et al., 2022, Xiong et al., 2022):

(2) $$THQ=\frac{CDI}{RfD\;or\;TDI}$$

In this equation, THQ is the target hazard quotient.

When THQ below than 1 value, the consumers are acceptable risk.

The carcinogenic risk of inorganic As was calculated below (FAO/WHO, 2011):

(3) CR = CDI × CSF

where, CR is carcinogenic risk and CSF, cancer slope factor. CSF for inorganic As is equal to 1.5 (mg/kg-d)^-1^. When CR is below 1.00E-6 value, cancer risk is (FAO/WHO, 2011).

## Results and discussion

### Level of PTEs in shrimps from the Mediterranean

**Fig. 1:**
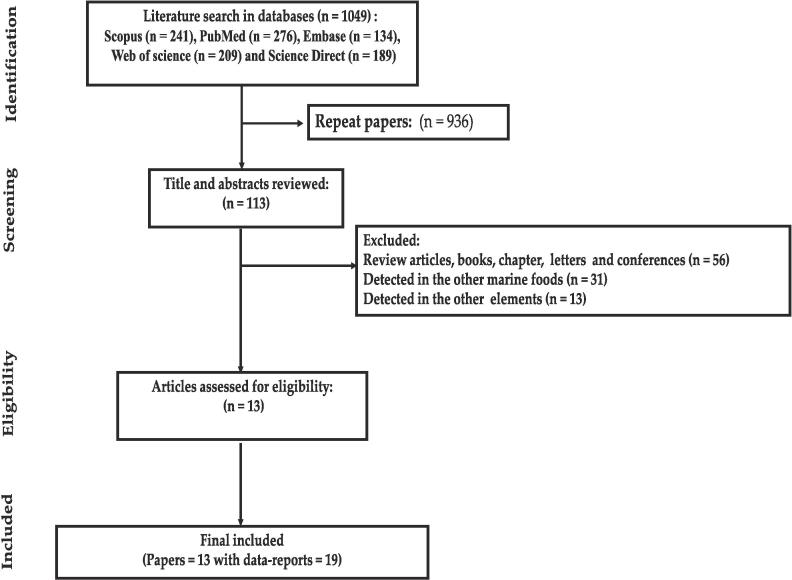
Process of selection of paper based on PRISMA.

**Fig. 2:**
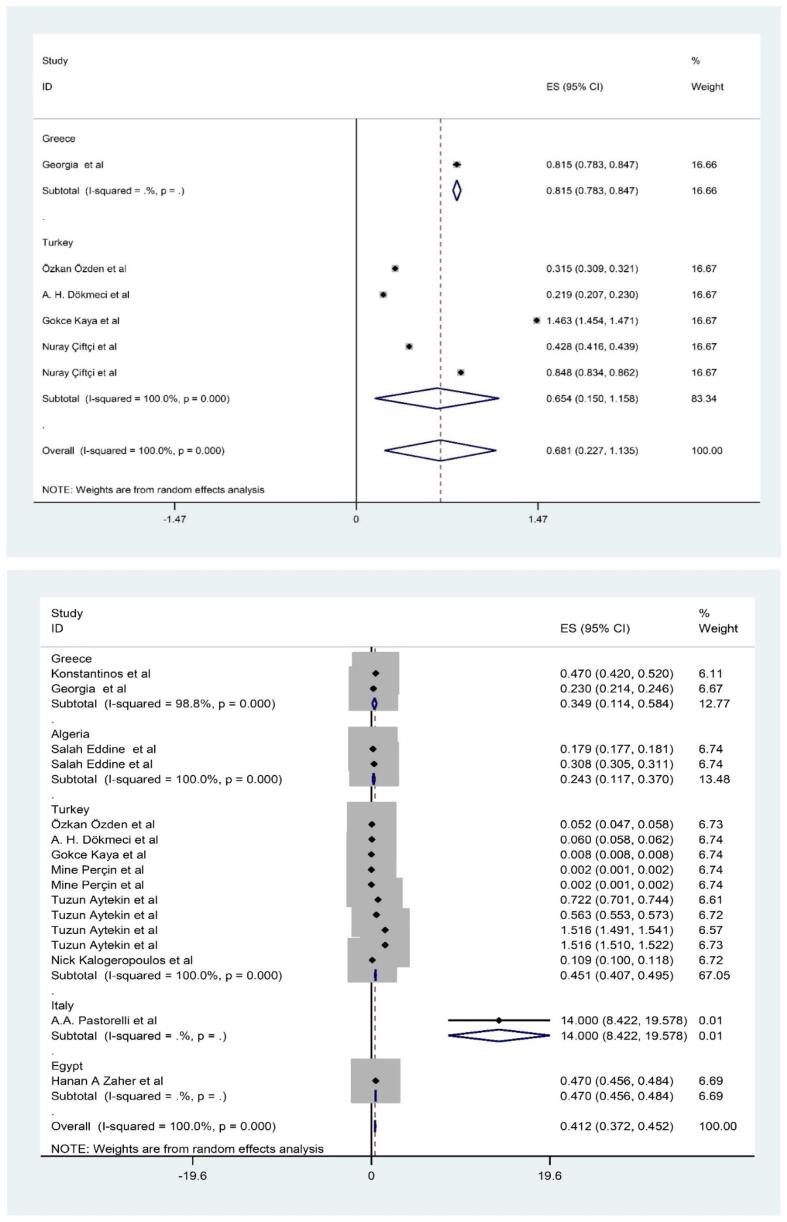
Meta-analysis concentration of inorganic As and Cd in filled of shrimps.

**Fig. 3:**
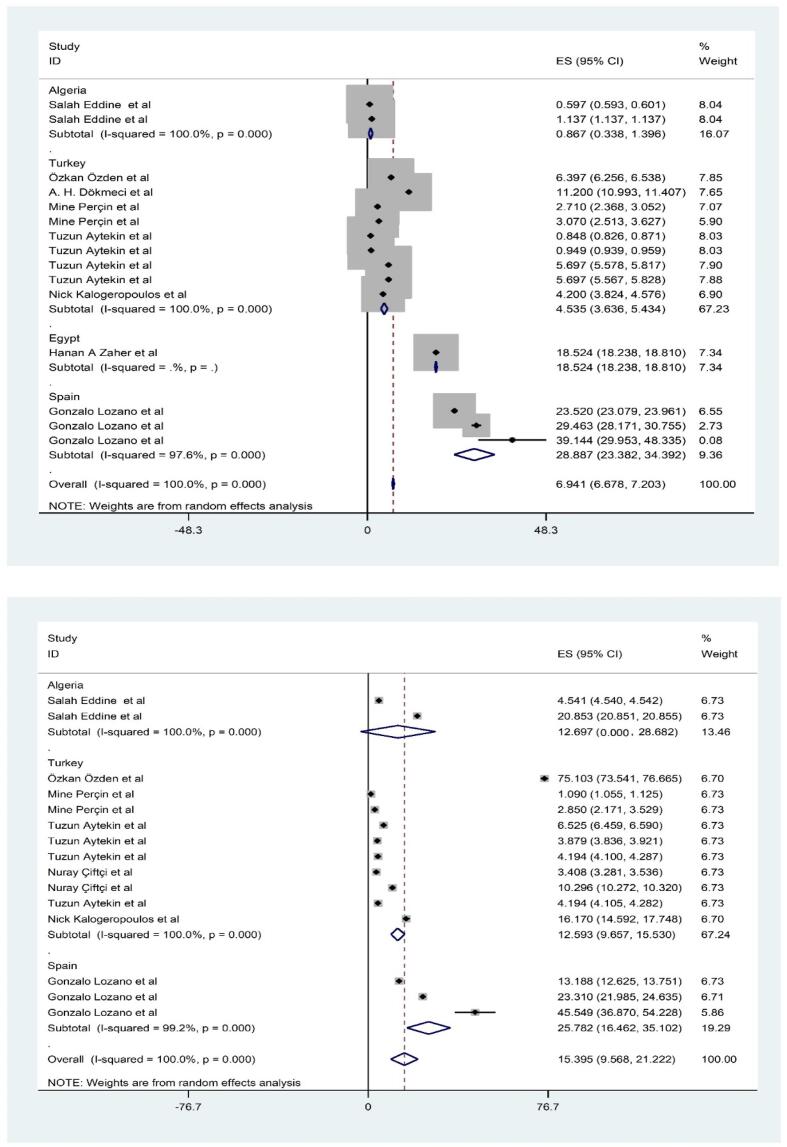
Meta-analysis concentration of inorganic Cu and Fe in filled of shrimps.

**Fig. 4:**
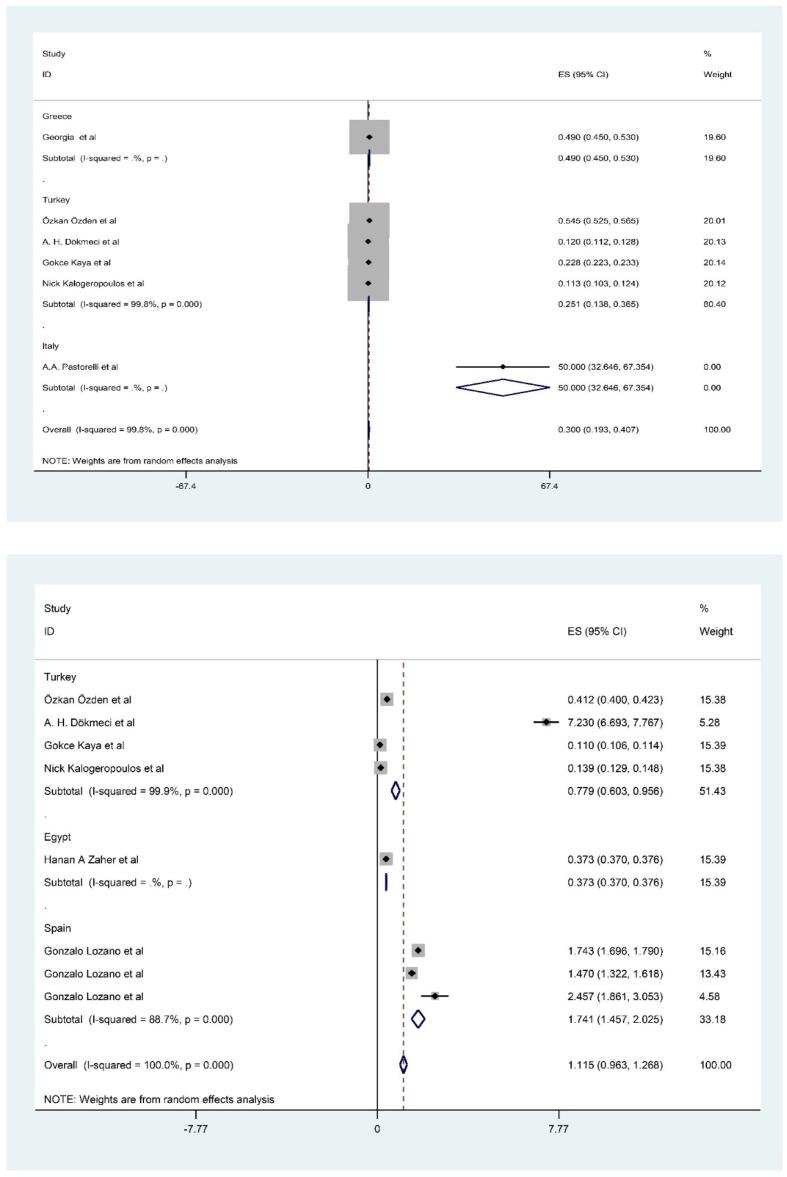
Meta-analysis concentration of inorganic methyl Hg and Ni in filled of shrimps.

**Fig. 5:**
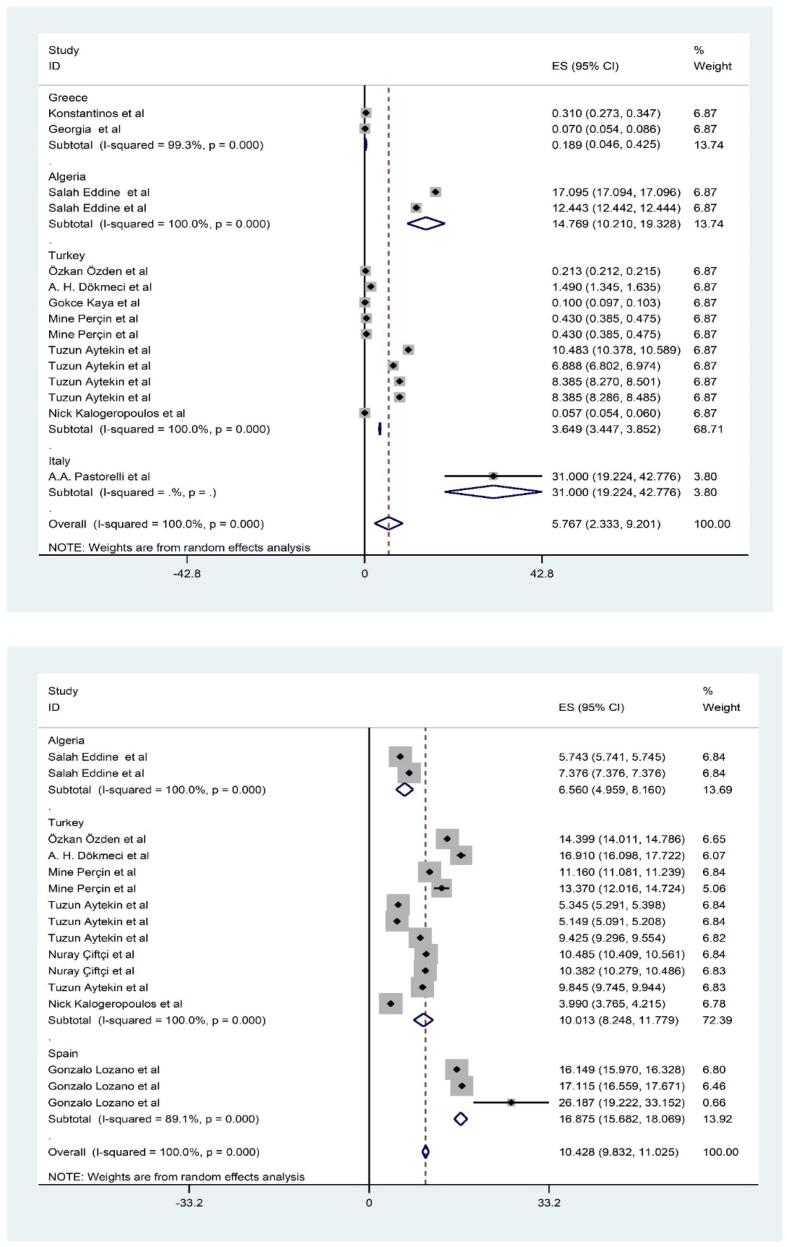
Meta-analysis concentration of inorganic Pb and Zn in filled of shrimps.

Thirteen papers with 19 data-reports included in out *meta*-analysis **(**Fig. 1 **and Appendix 2).** The rank order of PTEs based mean (pooled) level in fillet of shrimps was Fe (15.395 mg/kg-ww) > Zn (10.428 mg/kg-ww) > Cu (6.941 mg/kg-ww) Pb (5.7 mg/kg-ww) > Ni (1.115 mg/kg-ww) > As (0.681 mg/kg-ww) > Cd (0.412 mg/kg-ww) > Hg (0.300 mg/kg-ww) (Fig. 2, Fig. 3, Fig. 4, Fig. 5). The chemical quality of food can affect health in the long term (Geyik, 2023, Chaari, 2023, Thakaew & Chaiklangmuang, 2023). In this study, mean level of Fe, Zn and Cu as essential metals was higher than toxic metals group such as As, Cd and Hg (Talema, 2023). Essential metals exist in the body in small amounts but play an essential role in sustaining various physiological and metabolic processes occurring within living tissues, as enzymes structure and function (Xiao-Mei, 2023, Ghane et al., 2022, Khazaei et al., 2023). The mean level of PTEs in shrimps of our study was compared with those in other regions of the world. For example, Skordas et al (2022) reported the mean level Zn, Cu and Fe (5.7, 0.5 and 4.5 mg/kg) in *Parapenaus longirostris* of shrimps was lower than this study (Skordas et al., 2022). Oudainia et al (2023) indicated the mean level Fe (20.8 mg/kg) in *Plesionika edwardsii* of shrimps in Algeria was higher than this study, while level of Zn and Cu (7.3 and 1.1 mg/kg) was lower than this study (Oudainia et al., 2023). In other study conducted by Kalogeropoulos et al. (2012), which revealed substantial Fe contamination (56 mg/kg)in samples taken from the Mediterranean was (56 μg/g) that was higher than this study (Kalogeropoulos et al., 2012). They showed the distribution of the PTEs levels in in shrimps was Fe (75.2 mg/kg-ww) > Zn (69.8 mg/kg-ww) > Cu (10.9 mg/kg-ww) > Ni (0.64 mg/kg-ww) (Kalogeropoulos et al., 2012). In similar, Özden et al (2012) concluded the mean level Zn, Cu and Fe (14.3, 6.31 and 75.10 mg/kg) in *Parapenaus longirostris* of shrimps in Türkiye was higher than this study, while level of Ni (0.4 mg/kg) was lower than this study (Özden, 2010). Liu et al (2008) found mean level Cu (5.66 mg/kg) in Taihu aquatic organisms was higher than our study (W. Liu et al., 2008). On the other hand, the mean levels of Cu and Zn in shrimps were also found to be below the MRLs of 30 mg/kg and 100 mg/kg, respectively, which are established by the World Health Organization (National Research, 1989).

The concentration of PTEs in shrimps varies widely in different places (Zoghi et al., 2022, Xiao-Mei, Jin, Chao, Xiao-Jun, & Yuan, 2023). The obtained differences can be related to various natural and anthropogenic factors (Nie, Tu, Hu, Wu, & Zhou, 2023, Hu et al., 2022). The Mediterranean region has a complex geological history, and natural weathering of rocks and minerals can release trace metals into the environment. Shrimps and other aquatic organisms can accumulate these metals from water and sediments (Einolghozati et al., 2023, Cai, Zeng, & Li, 2023). Human activities can significantly contribute to PTEs levels in aquatic ecosystems (Onyeaka et al., 2022, Guzzi et al., 2021). Industries in the Mediterranean may release metals like Fe, Zn, and Cu into water bodies as part of their effluents. The use of fertilizers, pesticides, and other chemicals in agriculture can introduce metals into water bodies through runoff (Liu et al., 2008). In coastal waters, the presence of Fe, Mn, and Zn is primarily attributed to natural processes such as rock weathering, redox potential, soil erosion, and agricultural runoff. Additionally, external factors like the disposal of organic waste and sewage effluent contribute to their occurrence (Tabelin et al.). The breakdown of organic matter by microorganisms in sediment could lead to oxygen-deficient conditions at the pond bottom, converting soil Mn and Fe oxide into soluble forms (Jafarabadi, xxxx, Sabu, xxxx). The soluble Fe and Mn forms then enter surface waters through diffusion and turbulent mixing, resulting in higher levels compared to other metals (Scholtysik et al.). In this study, the levels of toxic metals such as Cd, Pb, Hg and As was investigated in fillet of shrimps in Mediterranean Sea. According to results, the ranking of toxic metals was Pb (5.7 mg/kg-ww) > As (0.681 mg/kg-ww) > Cd (0.412 mg/kg-ww) > Hg (0.300 mg/kg-ww) (Fig. 2, Fig. 3, Fig. 4, Fig. 5). The presence of these toxic elements in fillet of shrimps can cause problems for health and has caused major concerns for people and health policy makers in many countries worldwide. Regarding arsenic (As), it is present in various chemical forms in food, and organic As is generally less toxic than inorganic As (Ahmed et al.). This study specifically measured the level of inorganic As. In compared to our study, many previous studies investigated the mean level of toxic metals in fillet of shrimps. Kaya and Turkoglu (2017) indicated the mean level Pb, Hg and As (0.1 and 0.2 mg/kg) in *Penaeus semisulcatus* of shrimps in Türkiye was lower than this study, while level of As (1.41 mg/kg) was higher than this study (Kaya and Turkoglu, 2017). In other study conducted by Soultani et al. (2021), they revealed contamination of Pb and Cd (0.07 and 0.2 mg/kg) in samples taken from the Mediterranean was lower than this study, while level of Cd and As (0.4and 0.8 mg/kg) was higher than our study (Soultani et al., 2021). In similar, Kalogeropoulos et al (2012) concluded the mean level Pb, Hg and Cd (0.05, 0. 1 and 0.10 mg/kg) in *Parapenaus longirostris* of shrimps in Türkiye was higher than this study (Kalogeropoulos et al., 2012). The level of toxic PTEs like Cd and Hg in shrimps was observed to be below the Maximum Residual Limit (MRLs) of 0.5 mg/kg, as prescribed by the European Union Commission) (Council, 1989). In another research conducted in the Mediterranean region, it was found that arsenic levels varied between 0.05 and 0.86 mg/kg, with an average of 0.39 ± 0.04 mg/kg, while mercury levels ranged from 0.081 to 3.54 mg/kg, with a mean of 0.40 ± 0.04 mg/kg in the analyzed shrimps samples (Abd-Elghany et al., 2020). To assess the levels of PTEs pollution, the level of PTEs in shrimps was compared to data from previous global studies. Likewise, toxic PTEs including Cd, As, Hg, and Ni were also found to be lower compared to samples collected from Glenelg beach, Australia (Chakraborty and Owens, 2014), Xiangshusi coast in China (J. Fu et al., 2014), and Jinzhou Bay in China (Wang et al.). In the Persian Gulf, the levels of lead (Pb) and arsenic (As) found in the muscle tissues of locally sourced shrimps were noted to exceed the limits defined by the guidelines set by the Food and Agriculture Organization/World Health Organization (FAO/WHO) (Fakhri et al., 2018). According to reports, there was the wide variation between levels of toxic metals such as Cd, Pb, Hg and As from other studies and also our study. These differences may be due to metals feature, agricultural activities such as the types of fertilizers, processing technologies, industrial activities and mining, near to high-traffic roads, workshops and factories and as well as, storage condition are from effective factors in the observed differences (Mahmudiono et al., 2023).

### Health risk assessment

The non-carcinogenic risk of metals by the consumption of fillet of shrimps in Mediterranean Sea has been presented in (Table 1). Based on findings, THQ level in adults and children due to Cd and Pb in Italy was higher than 1 value (Table 1). THQ level in adults and children due to Cu, Ni, Fe, Zn and inorganic As was lower than 1 value (Table 1). CR due to inorganic As in Greece and Türkiye for adults and children was higher than 1E-6 value (Table 2). As, Cd, Hg, Pb, and Cu exhibit the greatest propensity for accumulating within fish tissues (Shahsavani et al., 2017). There were many agents affect the health risk such as the level of metals, ingestion rate of and consumption pattern of fillet of shrimps, body weight and exposure time (Sarkar et al., 2016).Consistent with our study, Yu et al (2020) in study conducted in China indicated (THQ) values for all PTEs remained below 1 (Yu et al., 2020). Notably, THQ Hg exhibited the highest value (THQ Hg = 0.458), still signifying the absence of potential health risks. However, it's essential to note that when individuals are exposed to multiple PTEs simultaneously, there's a possibility of combined or interactive effects, potentially leading to health concerns (Loaiza et al., 2018). Ezemonye et al (2019) in health risk assessment performed on samples from the Benin River revealed that THQ value of Zn was lower than 1, while Ni displayed the highest THQ (Ezemonye et al., 2019). In comparison, a previous study (Krishna et al., 2014) focusing on fish consumption in Andhra Pradesh, India, reported THQ values above 1, except for Cd, which remained below 1. Similarly, (Enuneku et al., 2014) conducted a risk assessment study for individuals in India and the United States, finding no health risk implications, be it carcinogenic (LCR < 10–6) or non-carcinogenic (THQ and TTHQ < 1), associated with the consumption of farmed shrimps. Although the shrimps species from various locations in the Khulna-Satkhira area of Bangladesh were deemed safe for consumption, the potential health risk linked to non-carcinogenic effects remains notably low, even with continuous consumption over a span of 30 years (Sarkar et al., 2016). Moreover, risk assessment studies carried out on Persian gulf also indicated significant health risks, whether carcinogenic (LCR < 10–6) or non-carcinogenic (THQ and TTHQ < 1), attributable to the consumption of shrimps (Fakhri et al., 2018).

**Table 1:** THQ of potential toxic element due to ingestion shrimps in Mediterranean Sea.

	Cd	Cu	Ni	Fe	Zn	Methyl Hg	Pb	Inorganic As
**Adults**								
Greece	4.23E-02					5.94E-01	6.37E-03	1.48E-04
Algeria	5.21E-03	4.65E-04		5.11E-04	4.69E-04		8.80E-02	
Turkey	1.40E-02	3.53E-03	2.21E-03	5.60E-04	1.04E-03	7.82E-02	3.15E-02	3.05E-05
Italy	**2.34E + 00**	7.73E-02			1.25E-02	**8.34E + 01**	**1.44E + 00**	
Egypt	6.83E-02		4.93E-03					
Spain		1.64E-01	3.52E-02	8.18E-03				
**Children**								
Greece	1.97E-01					**2.77E + 00**	2.97E-02	5.93E-05
Algeria	2.43E-02	2.17E-03		2.39E-03	2.19E-03		4.10E-01	
Turkey	6.55E-02	1.65E-02	1.03E-02	2.61E-03	4.85E-03	3.65E-01	1.47E-01	1.22E-05
Italy	**1.09E + 01**	3.61E-01			5.84E-02	**3.89E + 02**	**6.71E + 00**	
Egypt	3.19E-01		2.30E-02					
Spain		7.65E-01	1.64E-01	3.82E-02

**Table 2:** CR of inorganic As due to ingestion shrimps in Mediterranean Sea.

Adults	CR
Greece	1.48E-04
Turkey	3.05E-05
**Children**	
Greece	5.93E-05
Turkey	1.22E-05

## Conclusion

In this *meta*-analysis study, level of PTEs (Cd, Pb, As, Hg, Cu, Fe, Ni and Zn) was investigated fillet of shrimps in Mediterranean Sea using international databases until 20 July 2023. Results indicated that ranking of metals was Fe > Zn > Cu Pb > Ni > As > Cd > Hg. The level of metals in our study was lower than the permitted amount established by WHO. The according to risk assessment, THQ level in adults and children except Cd and Pb in Italy was lower than 1 value. It is well known that chemical characteristics of PTEs, geographic area, type of industries and active mines, type and number of chemical fertilizers used for agricultural, and product feeds have important roles on the level of PTEs in various fillets of shrimps in Mediterranean Sea. Also, it was suggested to study concentration of PTEs in the other shrimp species.

## CRediT authorship contribution statement

**Trias Mahmudiono:** Writing – original draft, Data curation, Conceptualization. **Zahra Esfandiari:** Writing – review & editing, Writing – original draft. **Ali Zare:** Writing – original draft. **Mohammadmahdi Sarkhoshkalat:** Writing – original draft, Resources, Conceptualization. **Fereshteh Mehri:** Writing – review & editing, Supervision, Conceptualization. **Yadolah Fakhri:** Writing – review & editing, Writing – original draft, Conceptualization.

## Declaration of competing interest

The authors declare that they have no known competing financial interests or personal relationships that could have appeared to influence the work reported in this paper.

## Data Availability

Data will be made available on request.
