# Supplementary material for: Concentration of potentially toxic elements in fillet shrimps of Mediterranean Sea: Systematic review, meta-analysis and health risk assessment
Source: Food Chem X. 2024 Feb 7;21:101206. doi: 10.1016/j.fochx.2024.101206 (PMC10876684; doi:10.1016/j.fochx.2024.101206)
Supplement: Supplementary data 1 [file mmc1.docx]

**Appendix 1.** Mean ingestion rate of shrimp in countries of around of Mediterranean Sea (OURWORLDINDATA, 2021) . By considering 17.5% (5-30 %) of the total consumption of seafood, the amount of shrimp consumption was obtained (Fan et al., 2022; report, 2020; Sharifian et al., 2022; Yu et al., 2020).

| **Country** | **Total Consumption Marin Food (kg/y)** | **Shrimp (mg/kg-d)** |
| --- | --- | --- |
| Greece | 21.54 | 10.327 |
| Algeria | 3.81 | 1.827 |
| Turkey | 5.53 | 2.651 |
| Italy | 29.64 | 14.211 |
| Egypt | 25.8 | 12.370 |
| Spain | 40.3 | 19.322 |

**Reference**

Fan, Y, KR Schneider, & PJ Sarnoski. (2022). Determining spoilage of whiteleg shrimp (Litopanaeus vannemei) during refrigerated storage using colorimetric strips. *Food Chemistry: X, 14*, 100263. <https://doi.org/https://doi.org/10.1016/j.fochx.2022.100263>.

OURWORLDINDATA. (2021). Mean ingestion rate of shrimp in countries of around of Mediterranean Sea <https://ourworldindata.org/grapher/fish-and-seafood-consumption-per-capita>*.*

report, e. (2020). <https://oceans-and-fisheries.ec.europa.eu/facts-and-figures/facts-and-figures-common-fisheries-policy/consumption_en>*.*

Sharifian, S, MS Mortazavi, & SLM Nozar. (2022). Health risk assessment of commercial fish and shrimp from the North Persian Gulf. *Journal of Trace Elements in Medicine and Biology, 72*, 127000.

Yu, B, X Wang, KF Dong, G Xiao, & D Ma. (2020). Heavy metal concentrations in aquatic organisms (fishes, shrimp and crabs) and health risk assessment in China. *Marine pollution bulletin, 159*, 111505. <https://doi.org/https://doi.org/10.1016/j.marpolbul.2020.111505>.
