# Supplementary material for: Concentration of potentially toxic elements in fillet shrimps of Mediterranean Sea: Systematic review, meta-analysis and health risk assessment
Source: Food Chem X. 2024 Feb 7;21:101206. doi: 10.1016/j.fochx.2024.101206 (PMC10876684; doi:10.1016/j.fochx.2024.101206)
Supplement: Supplementary data 2 [file mmc2.docx]

**Appendix 2.** Main characteristic included in our study

| **Species** | **Country** | **Sample size** | **AS** | **sd** | **Cd** | **sd** | **Hg** | **sd Hg** | **Pb** | **Ni** | **sd** | **fe** | **sd** | **Cu** | **sd** | **Zn** | **sd** |  | **Lod** | **Reference** |
| --- | --- | --- | --- | --- | --- | --- | --- | --- | --- | --- | --- | --- | --- | --- | --- | --- | --- | --- | --- | --- |
| Parapenaus longirostris | Greece | 10 |  |  | 0.4700 | 0.0800 |  |  | 0.3100 |  |  |  |  |  |  |  |  | Graphite Furnace Atomic Absorption Spectrometry | Not mentioned | ([Skordas et al., 2022](#_ENREF_11)) |
| Plesionika edwardsii (Male) | Algeria | 60 |  |  | 0.1790 | 0.0084 |  |  | 17.0950 |  |  | 4.5410 | 0.0031 | 0.5970 | 0.0170 | 5.7430 | 0.0082 | ICP AES | Not mentioned | ([Oudainia et al., 2023](#_ENREF_8)) |
| Plesionika edwardsii (Female) | Algeria | 60 |  |  | 0.3080 | 0.0117 |  |  | 12.4430 |  |  | 20.8530 | 0.0079 | 1.1370 | 0.0016 | 7.3760 | 0.0009 | ICP AES | Not mentioned | ([Oudainia et al., 2023](#_ENREF_8)) |
| Parapenaus longirostris | Turkey | 70 | 0.3150 | 0.0265 | 0.0525 | 0.0228 | 0.5450 | 0.0855 | 0.2135 | 0.4115 | 0.0478 | 75.1030 | 6.6670 | 6.3970 | 0.6015 | 14.3985 | 1.6543 | ICP-MS | 50 ppt for Hg - 2 ppt for Cd - 10 ppt for Pb - 20 ppt for Cu - 100 ppt for Zn - 50 ppt for Fe- 25 ppt for As - 30 ppt for Ni - | ([Özden, 2010](#_ENREF_9)) |
| Parapenaus longirostris | Turkey | 90 | 0.2185 | 0.0550 | 0.0600 | 0.0100 | 0.1200 | 0.0400 | 1.4900 | 7.2300 | 2.6000 |  |  | 11.2000 | 1.0000 | 16.9100 | 3.9300 | Flame Atomic Absorption Spectrophotometer | Not mentioned | ([Dökmeci et al., 2014](#_ENREF_3)) |
| Penaeus semisulcatus | Turkey | 32 | 1.4627 | 0.0237 | 0.0080 | 0.0010 | 0.2280 | 0.0160 | 0.1000 | 0.1100 | 0.0100 |  |  |  |  |  |  | ICP-MS | Ni 0.35، Cd 0.004 ، As 0.035،Pb 0.07،Hg 0.006 (μgkg−1) | ([Kaya et al., 2017](#_ENREF_5)) |
| Deep-water rose shrimp | Italy | 10 |  |  | 14.0000 | 9.0000 | 50.0000 | 28.0000 | 31.0000 |  |  |  |  |  |  |  |  | Q-ICP-MS | LOD should be lower thanone-tenth of the ML set by Commission Regulation 2006/1881/EC (Council of the European Union 2006 | ([Pastorelli et al., 2012](#_ENREF_10)) |
| Aristaeomorpha foliacea | Greece | 6 | 0.8150 | 0.0400 | 0.2300 | 0.0200 | 0.4900 | 0.0500 | 0.0700 |  |  |  |  |  |  |  |  | ICP-MS | Not mentioned | ([Soultani et al., 2021](#_ENREF_12)) |
| Parapenaus longirostris | Egypt | 50 |  |  | 0.4700 | 0.0505 |  |  |  | 0.3730 | 0.0100 |  |  | 18.5240 | 1.0300 |  |  | atomic absorption spectrophotometer | Ni: 0.05 ، cu: 0.005 mg/L | ([Zaher et al., 2021](#_ENREF_13)) |
| Aristaeomorpha foliacea | Turkey | 20 |  |  | 0.0017 | 0.0009 |  |  | 0.4300 |  |  | 1.0900 | 0.0800 | 2.7100 | 0.7800 | 11.1600 | 0.1800 | atomic absorption spectrophotometer | 2.5 ppb for Cd، 1 ppb for Pb | ([Olgunoğlu et al., 2015](#_ENREF_7)) |
| Aristaeomorpha foliacea | Turkey | 20 |  |  | 0.0017 | 0.0009 |  |  | 0.4300 |  |  | 2.8500 | 1.5500 | 3.0700 | 1.2700 | 13.3700 | 3.0900 | atomic absorption spectrophotometer | 2.5 ppb for Cd، 1 ppb for Pb | ([Olgunoğlu et al., 2015](#_ENREF_7)) |
| Solea solea | Turkey | 42 |  |  | 0.7224 | 0.0714 |  |  | 10.4832 |  |  | 6.5247 | 0.2163 | 0.8484 | 0.0746 | 5.3445 | 0.1785 | ICP-MS | 0.028 ppm | ([Aytekin et al., 2019](#_ENREF_1)) |
| Sparus aurata | Turkey | 40 |  |  | 0.5628 | 0.0315 |  |  | 6.8880 |  |  | 3.8787 | 0.1365 | 0.9492 | 0.0315 | 5.1492 | 0.1890 | ICP-MS | 0.028 ppm | ([Aytekin et al., 2019](#_ENREF_1)) |
| Penaeus semisulcatus | Turkey | 44 |  |  | 1.5162 | 0.0840 |  |  | 8.3853 |  |  | 4.1937 | 0.3171 | 5.6973 | 0.4053 | 9.4248 | 0.4372 | ICP-MS | 0.028 ppm | ([Aytekin et al., 2019](#_ENREF_1)) |
| Penaeus semisulcatus | Turkey | 40 | 0.4277 | 0.0366 |  |  |  |  |  |  |  | 3.4083 | 0.4116 |  |  | 10.4853 | 0.2457 | ICP-MS | Not mentioned | ([Çiftçi et al., 2021](#_ENREF_2)) |
| Penaeus semisulcatus | Turkey | 40 | 0.8476 | 0.0452 |  |  |  |  |  |  |  | 10.2963 | 0.0777 |  |  | 10.3824 | 0.3339 | ICP-MS | Not mentioned | ([Çiftçi et al., 2021](#_ENREF_2)) |
| Penaeus semisulcatus | Turkey | 44 |  |  | 1.5162 | 0.0210 |  |  | 8.3853 |  |  | 4.1937 | 0.3003 | 5.6973 | 0.4410 | 9.8448 | 0.3360 | ICP-MS | 0.028 ppm | ([Aytekin et al., 2019](#_ENREF_1)) |
| Parapenaus longirostris | Turkey | 30 |  |  | 0.1092 | 0.0246 | 0.1134 | 0.0284 | 0.0567 | 0.1386 | 0.0273 | 16.1700 | 4.4100 | 4.2000 | 1.0500 | 3.9900 | 0.6300 | flame atomic absorption spectrometry | 2.6 ng/g for Cd, 0.05 lg/g for Cr, 0.06 lg/g for Cu, 6.0 lg/g for Fe, 7.5 ng/g for Hg, 0.08 lg/g for Ni, 0.04 lg/g for Pb and 1.0 lg/g for Zn | ([Kalogeropoulos et al., 2012](#_ENREF_4)) |
| Palaemon elegans | Spain | 1525 |  |  |  |  |  |  |  | 1.7430 | 0.9450 | 13.1880 | 11.2140 | 23.5200 | 8.7780 | 16.1490 | 3.5700 | ICP-MS | Not mentioned | ([Lozano et al., 2010](#_ENREF_6)) |
| Palaemon serratus | Spain | 100 |  |  |  |  |  |  |  | 1.4700 | 0.7560 | 23.3100 | 6.7620 | 29.4630 | 6.5940 | 17.1150 | 2.8350 | ICP-MS | Not mentioned | ([Lozano et al., 2010](#_ENREF_6)) |
| Palaemon adspersus | Spain | 24 |  |  |  |  |  |  |  | 2.4570 | 1.4910 | 45.5490 | 21.6930 | 39.1440 | 22.9740 | 26.1870 | 17.4090 | ICP-MS | Not mentioned | ([Lozano et al., 2010](#_ENREF_6)) |

**Reference**

Aytekin, T, D Kargın, HY Çoğun, Ö Temiz, HS Varkal, & F Kargın. (2019). Accumulation and health risk assessment of heavy metals in tissues of the shrimp and fish species from the Yumurtalik coast of Iskenderun Gulf, Turkey. *Heliyon, 5*(8).

Çiftçi, N, D Ayas, & M Bakan. (2021). The comparison of heavy metal level in surface water, sediment and biota sampled from the polluted and unpolluted sites in the northeastern mediterranean sea. *Thalassas: An International Journal of Marine Sciences, 37*(1), 319-330.

Dökmeci, AH, T Yildiz, A Ongen, & N Sivri. (2014). Heavy metal concentration in deepwater rose shrimp species (Parapenaeus longirostris, Lucas, 1846) collected from the Marmara Sea Coast in Tekirdağ. *Environmental Monitoring and Assessment, 186*, 2449-2454.

Kalogeropoulos, N, S Karavoltsos, A Sakellari, S Avramidou, M Dassenakis, & M Scoullos. (2012). Heavy metals in raw, fried and grilled Mediterranean finfish and shellfish. *Food and Chemical Toxicology, 50*(10), 3702-3708.

Kaya, G, & S Turkoglu. (2017). Bioaccumulation of heavy metals in various tissues of some fish species and green tiger shrimp (Penaeus semisulcatus) from İskenderun Bay, Turkey, and risk assessment for human health. *Biological Trace Element Research, 180*, 314-326.

Lozano, G, E Herraiz, A Hardisson, AJ Gutiérrez, D González-Weller, & C Rubio. (2010). Heavy and trace metal concentrations in three rockpool shrimp species (Palaemon elegans, Palaemon adspersus and Palaemon serratus) from Tenerife (Canary Islands). *Environmental Monitoring and Assessment, 168*, 451-460.

Olgunoğlu, MP, İA Olgunoğlu, & YK Bayhan. (2015). Heavy Metal Concentrations (Cd, Pb, Cu, Zn, Fe) in Giant Red Shrimp (Aristaeomorpha foliacea Risso 1827) from the Mediterranean Sea. *Polish Journal of Environmental Studies, 24*(2).

Oudainia, SE, F Derbal, O Alik, & N Bourehail. (2023). Seasonal and Sexual Variations of Trace-Metal Elements (Cd, Pb, Cu, Zn, Fe) in Muscle and Eggs of the Deep-Water Pandalid Shrimp Plesionika edwardsii (Brandt 1851) from Northeast Algeria. *Thalassas: An International Journal of Marine Sciences, 39*(1), 263-271.

Özden, Ö. (2010). Seasonal differences in the trace metal and macrominerals in shrimp (Parapenaus longirostris) from Marmara Sea. *Environmental Monitoring and Assessment, 162*, 191-199.

Pastorelli, AA, M Baldini, P Stacchini, G Baldini, S Morelli, E Sagratella, S Zaza, & S Ciardullo. (2012). Human exposure to lead, cadmium and mercury through fish and seafood product consumption in Italy: a pilot evaluation. *Food Additives & Contaminants: Part A, 29*(12), 1913-1921.

Skordas, K, A Lolas, C Gounari, K Georgiou, N Neofitou, & D Vafidis. (2022). Trace Element Content and Potential Human Health Risk from Consumption of the Deep-water Rose Shrimp Parapenaeus longirostris (Crustacea: Decapoda) from Pagasitikos Gulf, Greece. *Journal of Chemical Health Risks, 12*(4), 675-683.

Soultani, G, V Sele, RR Rasmussen, I Pasias, E Stathopoulou, NS Thomaidis, VJ Sinanoglou, & JJ Sloth. (2021). Elements of toxicological concern and the arsenolipids’ profile in the giant-red Mediterranean shrimp, Aristaeomorpha foliacea. *Journal of Food Composition and Analysis, 97*, 103786.

Zaher, HA, AH Mohamed, SE Hamed, & A El-Khateeb. (2021). Risk Assessment of Heavy Metal Bioaccumulation in Raw Crab and Prawn Flesh Marketed in Egypt. *Journal of Human Environment and Health Promotion, 7*(1), 6-14.
